# Iranians’ Perspective to Cosmetic Surgery: A Thematic Content Analysis for the Reasons

**DOI:** 10.29252/wjps.8.1.69

**Published:** 2019-01

**Authors:** Nahid Mozaffari Niya, Majid Kazemi, Farrokh Abazari, Fazlollah Ahmadi

**Affiliations:** 1 Nursing Research Center, Razi Faculty of Nursing and Midwifery, Kerman University of Medical Sciences, Kerman, Iran;; 2 Nursing Department, Rafsanjan University of Medical Sciences, Rafsanjan, Kerman, Iran;; 3 Nursing Department, Faculty of Medical Sciences, Tarbiat Modares University, Tehran, Iran

**Keywords:** Face, Destiny, Content analysis, Decision, Cosmetic surgery, Iran

## Abstract

**BACKGROUND:**

Currently, becoming beautiful is a value among Iranian society, although experts have suggested several complications of cosmetic surgery, but decision to have these surgeries has been dramatically increased in recent years. This increase has imposed high workload and costs on the health care system of the country. This study was conducted to explore the reasons why people perform cosmetic surgery in an Iranian context.

**METHODS:**

Twenty-one subjects from both sexes who were 22-52 year-old and had undergone face plastic surgery between 1-5 years ago were enrolled and semi-structured data was collected via open interviews, while qualitative content were analyzed utilizing conventional analysis methods. Data analysis considered the theme “my face, my destiny” which contained 2 subcategories of “obtaining a chance for a better life “and” obtaining acceptance in society”.

**RESULTS:**

The participants considered cosmetic surgery as a blessing from God that played a major role in their future, so they accepted the risks of the surgery.

**CONCLUSION:**

Understanding experiences of these individuals may help health care team particularly nurses to consult them and other individuals who might think about doing cosmetic surgery through education to help them make a better decision for to do cosmetic surgery.

## INTRODUCTION

One of the essential needs of man in modern times is “being seen beautiful”.^1^ Modern society has determined a high competitive standard for beauty.^2^ Therefore, becoming beautiful based on defined standards can frustrate people who are not beautiful,^1^ and changes in the modern society have caused people to draw more attention to their bodies and how to manage and control it in the modern consumer society. One of the behaviors observed in most societies is cosmetic surgery.^3^ Accordingly, the willingness of men and women who are interested in having cosmetic surgery has increased dramatically.^4^ Cosmetic surgery is a surgical procedure with the aim of improving the appearance by increasing physical features.^5^ According to the reports of the American Society of Plastic Surgeons, cosmetic surgical procedures in the United States have been increased to 98% from 2000 to 2012 while 72% of these surgeries have been performed because of beauty and to improve the appearance and increase self-confidence.^6^

At the moment, becoming beautiful is a value for the current Iranian society that has accelerated competition for becoming beautiful.^1^ According to global studies on cosmetic surgery, Iran is the country with the highest rate of cosmetic procedures, as reportedly, in terms of the ratio of people who carried out cosmetic surgeries rather than the whole population. In 2013, Iran was ranked first in the world, while more than 175,000 surgical and non-surgery operations have been carried out in Iran.^7^ In today’s society of Iran, women give more importance to beauty, although many men may attempt to perform cosmetic surgery in order to improve their appearance.^1^

Researchers have shown that there are two types of motivation why people perform cosmetic surgeries including social and intrapersonal factors.^8^ Some studies have revealed that intrapersonal factors such as self-assessment, attractiveness,^9^^,^^10^ appearance-based rejection sensitivity,^11^^,^^12^ and negative body image^13^^-^^16^ are the primary stimulants for cosmetic surgery. Socio-cultural factors can also increase their interest in cosmetic surgery, because people are increasingly exposed to cosmetic surgery via social networks.^16^^-^^20^ Not only media but also parents and friends may also transfer messages about the importance of physical appearance and the applied strategies for increasing the attractiveness.^17^^-^^21^ These external and internal pressures which are due to the physical appearance and body image lead to feelings of inadequacy and anxiety in women.^22^^,^^23^

Women are more concerned about how other people in the community may judge them based on their physical appearance.^2^ According to socio-cultural perspective, people judge others based on their physical appearance and behave differently towards them, so that attractive people are often more favorable than unattractive people.^24^ Similarly, another study showed that those who have less facial attraction are less favorable compared with those that have higher facial attraction.^25^ Most cosmetic surgeries are carried out for reasons and hope for higher satisfaction from their appearance, but the main motivation for implementing cosmetic surgeries is to improve their mental performance.^26^

Though performing cosmetic surgeries can be helpful in terms of both physical and psychological health aspects, but if it is not properly implemented, it can bring many dangers for the consumers.^27^ Unofficial statistics have shown that during the year, almost 120 people were victims of these surgeries.^28^ On the other hand, excessive surgeries result in financial burden on the economy of the community and the health care system. It has been reported that Iranian men spend more than 50 million dollars annually for rhinoplastic surgeries, while they spend almost two times of that amount on Botox. However, women accounted for the majority of applicants for cosmetic surgeries,^29^ so this issue should also be considered as a medical complication.

According to an undertaken research, clinical services providers played a crucial role in decision-making and helping people to carry out their actions.^30^ When cosmetic surgery is carried out only for cosmetic reasons, clinical services providers should be particularly vigilant in assessing opportunities for surgery, because there are physical and psychological risks involved.^31^ Currently, increase in the number of applicants for cosmetic surgery has created a lot of questions and the fundamental question that is always asked despite the risks involved in it is that do patients referred for cosmetic surgery consider the motivations and consequences?

According to the mentioned points, cosmetic surgery is a multi-faceted phenomenon that is associated with mental and social parameters and many quantitative studies have revealed the reasons for cosmetic surgery, while there are few numbers of qualitative researches that reveal attitude and personal experiences of individuals. Therefore, the aim of this study was to recognize the motivations considered by men and women for cosmetic surgeries and also explain their experience of cosmetic surgery based on their own narration.

## MATERIALS AND METHODS

This study identified the factors influencing the decision to perform deep facial cosmetic surgeries. The study was carried out in West of Tehran, Iran between the year 2014 and 2015 utilizing qualitative content analysis. Qualitative content analysis as a qualitative research method and also a qualitative data analysis method was used.^32^ Content analysis method as a subjective interpretation of the content of the text that was carried out by the classification codes and identification of themes regularly.^33^ Content analysis was appropriate for this research because it is a good method of analysis that can easily accommodate a large amount of data.^32^

After obtaining the necessary permissions, the researchers referred to the Medical Records Department of Cosmetic Surgery Center, then the lists of the names of people who had undergone surgery at the center, and 21 participants who had cosmetic surgeries (7 men and 14 women) between one and five years ago were enrolled. Inclusion criteria included cosmetic surgery carried out under general anesthesia, at least three months after the surgery, possibility of accessing their addresses and phone numbers, completely vigilant at the time of data collection and willing to participate in the study. This research tried to qualify individuals with maximum diversity in terms of age, sex and type of cosmetic surgery.

The study was adopted by the Medical Research Ethics Committee at the School of Nursing and Midwifery, Kerman University of Medical Sciences Number (K.93/241, 2014/ 10/ 04). Informed consent was obtained from all participants. Participants were assured that they will remain anonymous and they are free to stop the interview at any time; in addition, they can refuse to answer any question. Data was collected via semi-structured interviews which were implemented through face to face interview techniques by a member of the research team (team members, a professor, associate professor and two PhD holders). First, the researchers explained the objectives of the study to participants. The interviews were carried out mainly at work, home while few participants were in the park. Interviews lasted between 45 to 90 min, averaging 65 min. Data were collected from 21 interviews.

At the beginning of the interview, the open-ended questions were asked in order to encourage participants on which subjects they could freely speak about personal experiences. Examples of questions asked were as follows: how were you feeling before the surgery? Why did you decide to perform this surgery? As well as considering the experiences that the participants expressed, other probing questions were asked depending on each participant and were suggested to fully understand the experiences. In order to increase the depth of the interviews, probing questions like; what do you mean? Explain and give an example, were used.

Data was analyzed with the approach of Landman and Granhaym.^34^ This means that all interviews were recorded with an MP 3 player device. Interviews were listened several times and were typed using verbatim and the data were studied in order to better understand the meaning, units in the interviews were identified and initial codes were extracted. Codes were classified based on similarities in themes and sub-themes. When themes were emerged and the data was saturated, interviews ended. The main theme including several themes and sub-themes was extracted. The accuracy and robustness of data (validity, reliability, and the ability to transfer confirmation) were examined utilizing the criteria proposed by Cuba and Lincoln.^35^

For data validation, interaction and closeness with participants as well as peer review and comparison was fixed. Data reliability was assessed by evaluation experts and appeals were made by the participants and outside observers. The researchers also tried to avoid previous judgments and to achieve adequate compliance, their previous beliefs were ignored. To transfer the research results to other people who were eligible for the study, findings were presented to them in order to approve the extracted concepts with their experiences. Drawing public attention and drawing attention of the opposite sex were introduced as sub-categories of “obtaining acceptance in society” and finding the right person to marry, finding job and high social status, and finding living position with the knowledge given by God were classified as sub-categories of “obtaining a chance for a better life”. The main categories and sub-categories were listed in Table 1.

**Table 1:** Main categories and sub-categories of my face, my destiny theme

**Themes **	**Main categories**	**Sub-categories**
My face, my destiny	Obtaining a chance for a better life	-Finding the right person to marry -Finding job and high social status -Finding living position with the knowledge given by God
	Obtaining acceptance in society	-Drawing public attention -Drawing attention of the opposite sex

## RESULTS

Thirteen (13) participants were between 22-30 years of age and the rest were over 30 years. Eleven (11) of them were employed and the rest were housewives or students. Five (5) of the participants had diplomas and the rest had university education, 9 of the participants were single, 11 of them were married and 1 of them was divorced. Trying to obtain a chance for a better life was one of the reasons for cosmetic surgery. Marriage, employment, and success were the most important reasons for men and women, especially women who were seeking for social opportunities in the community and cosmetic surgery gave them that chance in this situation. Most interviewees believed that beauty was the main criterion for marriage in this era. Hence everyone must be beautiful to be popular. 

Women were especially forced to have the right partner, in the face of competition; there were different ways to enhance their beauty and one of them was cosmetic surgery. “I feel if I had no cosmetic surgery, maybe I could not marry with a suitable case” (A 24-year old woman, married and student). Another participant said that: “Many of us, whether boy or girl performed cosmetic surgeries to find someone better for marriage” (A 22-year old man, single, and self-employed). A participant in response to the question that: “what do you want to achieve from cosmetic surgery? She said: “Finding a suitable case for marriage, to increase my suitors, so that I can choose the best one to marry” (A 24-year old girl, single and student). 

Some believe that since in our society, girls are more selected, then they should be completely flawless, meaning that evaluative look lead them to pay attention to their facial beauty. *“*In our society, a girl can never propose to a man, so we have to wait, and it should be totally an ideal case for them to come to us, for example, we have to carry out a series of surgeries to find our favorable person” (A 26-year old girl, single and graduate student). The quest for achieving a perfect job position is another factor that tends to motivate them to perform cosmetic surgery. “It was important for me to have a good face. because when you have surgery, your self-confidence is raised and influences the job that we want to have, or maybe you want to have a very high social position so you should have a good face” (A 22-year old man, single, and self-employed). “Now, even going to work and finding jobs are related to women beauty in our society. I went to many places to look for job and I saw they only considered my beauty not veils and my behavior. They just looked at you that you are beautiful or not, then they let you enter the workplace” (a 26-year-old woman, single and student).

Some participants believe that cosmetic surgery is a blessing from God and think it is a science that has been given to human, therefore performing these surgeries has no problem and sometimes they are necessary. “Cosmetic surgery is a science... if it is interfering with God’s work, then no operation should be implemented and no one should be treated. There are many tools for saving the lives of people, whenever there is a problem, a solution should be found. A lot of people came to the world with problems of physical appearance, then why should not cosmetic surgery be used to solve this problem?” (A 42-year old woman, married and practitioner). “I say that cosmetic surgery is a blessing that God gave us... If God did not want it, then he should not have given this knowledge ... If he gave this knowledge, then humans can change him/herself a little for more beauty...” (A 22-year old man, married, diploma and soldier).

According to the interviewees, they were seeking to have two types of attention by performing cosmetic surgery including drawing public attention and drawing the attention of the opposite sex. When the body and the appearance of a person is in violation of the appearance, weight, height and shape which are common social status, then the people might be noticed since they lack social position. Therefore, members of a social group may convince and force people to be socially acceptable through judgment, reward, and punishment of people. Acceptance in society is another motivation for performing cosmetic surgery. “When I see someone who has a better appearance, I feel the person is more acceptable in the society, when I see, I want to marry or I am looking for a job, people look at the if I am beautiful or not, then I said to myself, why not perform cosmetic surgery?” (A 22-year old man, single, and self-employed). 

“When I see that women who have undergone cosmetic surgery are of most interest to the community, then I said to myself, why do not I do it?” (A 24-year old woman, married and student). The “face” is used as the most exposed body part in social communications. Facial attractiveness makes predictable differences in general and specific communications, while the impacts and attractiveness of faces can be changed and adjusted in combination with other features, and status changes. For these people, cosmetic surgeries influence communications and draws public attention to them. “As soon as you do cosmetic surgery, the group of people who live with you in the workplace will change their behavior towards you, they somehow deal with you in a way I do not understand, it seems as if you did not have that character before, after surgery they all behave well towards you and more acceptable to you” (A 26-year old girl, single, and practitioner).

Women and men are socialized in such a way that they see themselves through the eyes of the opposite sex. They have been socialized in such a way that they consider the view of opposite sex about themselves. “I always thought I can attract a man, ... be beautiful in his eyes..., when you are more pretty, better men will give marriage proposal to you and have a better position in the society” (A 24-year old girl, single and student). “The opposite sex certainly had an impact… never a man does not catch own beauty for his own sake, and just does it for the opposite sex, especially in Iran” (A 24-year old man, married, diploma and soldier).

Someone introduced failing to attract the opposite sex as motivation to perform cosmetic surgery. “I was a 19-20 years old girl. At this age, the girls want to draw the attention of the opposite sex. When I went out I felt men do not pay any attention to me. I always thought it may be because of my nose that caused to be less my beauty. I’ m not saying I need them to look at me, but every girl feels that she wants to attract a man to herself “ (a 24-year old girl, single and student). Therefore, it can be said that pretty face as a capital in this context for women and men, gives them the opportunity to pursue a different approach for identification and desirable lifestyle and finally use these tools to convert this capital into opportunities and advantages in personal and social life (attention, popularity, position, concessions in the marriage etc.).

## DISCUSSION

In this study, it was demonstrated that “obtaining a chance for a better life” and “obtaining acceptance in society “ were the reasons why people decided to perform cosmetic surgery. The experiences of participants indicated that they were trying to achieve opportunities and privileges and finally socio-economic and social success by performing cosmetic surgery. They think obtaining opportunities such as marriage and employment were related to the use of cosmetic surgery. Most people expect that cosmetic surgeries gave them a young and beautiful look, and increased their chances of obtaining decent jobs, income, and better marriage. The results revealed that if people are not willing to take the risk of cosmetic surgery, then they are more likely to lose their ideal career opportunities. On the other hand, they believe that cosmetic surgery is a blessing from God and believe that it is a science given to humans, accordingly interpreted that using this science is essential in their life and also they believe that a person may lose a good chance in her life due to defects or physical appearance.

Since the social and professional opportunities are unevenly distributed, then some people may not be beautiful in terms of culture and fail to achieve the necessary facilities, and therefore, be disappointed.^1^ In fact, women have the expectations towards a desirable social position which lead them to attempt entertainments including superficial and cosmetic surgery.^36^ Cosmetic surgery is an opportunity given to people so that they rebuild their body as dictated by the media and society. Making such an identity is a dream that drove the people to accept the risk of cosmetic surgery.^1^ Accordingly, having a cosmetic surgery regardless of the outcome is a determinant priority in their lives.

It has been suggested that physical attractiveness plays a crucial role in relationships. Attractive people receive more job offers than unattractive applicants.^37^ It has also been shown that beauty is a product that can be sold, and women who do not search for it cannot successfully achieve this product socially.^38^ In another study using meta-analysis techniques, it was found that attractive people are more likely to be evaluated as qualified and competent professional. In a study carried out in 26 countries worldwide, both men and women introduced attractive face as one of the most important criteria for selecting a wife.^39^ In a qualitative research on people who had the desire to perform cosmetic surgery, it was demonstrated that they could act with social and emotional benefits to their own advantage.^40^

It has been reported that many social benefits of a beautiful young appearance, such as employment and romantic prospects may be motivated more by women using this method.^41^^,^^42^ In an ethnographic study on Brazilian women, it was shown that body dissatisfaction, achieving acceptance of partner, fear of “being replaced” by a more attractive woman, and the desire to improve the chances of achievement and friends were the main reasons for performing cosmetic surgeries.^43^ It has also been stated that achieving attractive face to acquire employment and social situations increased the chances of dating and finding wife and were factors involved in performing cosmetic surgery.^44^

The mentioned reasons have also been introduced in the present study as priorities in terms of audience perspective. Concerns about their appearance lead women to accept the option of performing cosmetic surgery as a means to increase their self-confidence and increase their social and professional potential.^8^^,^^45^^,^^46^ Some studies in Iran have demonstrated that cosmetic surgery may improve a person’s self-esteem, sexual attractiveness, so that through cosmetic surgery, the individual in society will be accepted and allowed to move upward economically and socially.^1^

Several studies have revealed that people take beauty as a factor that can hinder them from having a successful social role in their interactions and therefore make all attempts in order to be seen and accepted and can also make their body form similar to the social accepted pattern. They want to present themselves for being seen and draw others attention especially the attention of the opposite sex. According to previous findings, the growing importance of attractiveness and its importance in success, lead to increased social pressure to be attractive, and sometimes it seems that social acceptance and personal success depends to a large extent on beauty,^47^ because appearance is important, and it is the key factor for success in life.^48^

In another study, it was concluded that surgery is a tool for being more attractive than others and it had social rewards.^49^ Similarly, it was demonstrated that cosmetic surgery is considered by some people as a way of obtaining the expectations of other people and draw their attention.^50^ In a research in Iran, it was revealed that there was a significant relationship between social factors such as finding the right partner, social recognition by others, finding better jobs, job promotion and trend toward cosmetic surgery in women and the tendency to have cosmetic surgery has become a fashion and it was considered in determining social status.^51^ In a qualitative research aimed at evaluating the subjective reasons why Tehran citizens resorted to cosmetic surgery , social acceptance was identified as the reason they perform cosmetic surgery.^52^

In this study, face cosmetic surgery was taken into consideration as a group and community function in the context of urban life in Tehran, Iran. In the new Iranian society, the importance of appearance has increased as a measure of value because, appearance and beauty were considered as selection criteria for desirability and were also even better tools to succeed and enter into higher societies. The findings of the present research demonstrated that based on the participants’ experiences, beauty and having it, were important for people more than anything else, and most people like to look good and be involved in community because face was the most important aspect in the person and individuals felt that beautiful face helped them to have better social presence and also be more successful in their interactions. Otherwise, they may be rejected by society and jobs and opportunities for a better life.

It can be said that the people resort to cosmetic surgery in order to show their identity and dignity and promote the different stages of life, the continuation of this success, popularity, better positioning in marriage and other concessions. It seems that the body attractions especially facial beauty, lead to human freedom and choice to their fate and chance to a better life and this can be a reason why Iranian men and women crave for cosmetic surgery despite the risks involved and to change their appearance for encouragement. 

However, the study results provided valuable insight to healthcare providers. Understanding experiences of these individuals may help health care team particularly nurses to consult them and other individuals who might think about doing cosmetic surgery through education to help them make a better decision for to do cosmetic surgery. In this study, despite the mechanisms applied to enhance the accuracy, such as participation in the study with maximum variation had some limitations. The field was limited to a specific geographic location, while other people may experience other things than the ones mentioned in this study. It is therefore recommended to conduct other studies in other areas. 
